# COVID-19-Related Diplopia and Its Treatment

**DOI:** 10.3390/medicina61040626

**Published:** 2025-03-28

**Authors:** Shannon Patricia, Antonia Kartika, Irma Melyani Puspitasari

**Affiliations:** 1 Bachelor Program in Pharmacy, Faculty of Pharmacy, Universitas Padjadjaran, Sumedang 45363, West Java, Indonesia; shannon21001@mail.unpad.ac.id; 2 National Eye Center Cicendo Eye Hospital, Bandung 40117, West Java, Indonesia; antonia_kartika@yahoo.com; 3 Ophthalmology Department, Faculty of Medicine, Universitas Padjadjaran, Sumedang 45363, West Java, Indonesia; 4 Department of Pharmacology and Clinical Pharmacy, Faculty of Pharmacy, Universitas Padjadjaran, Sumedang 45363, West Java, Indonesia; 5 Center of Excellence for Pharmaceutical Care Innovation (PHARCI), Universitas Padjadjaran, Sumedang 45363, West Java, Indonesia

**Keywords:** COVID-19, diplopia, treatment, corticosteroids, vaccine

## Abstract

*Background and Objectives*: The effects of COVID-19 disease can manifest and cause eye complications, one of which is diplopia. Diplopia is a medical condition that makes one object appear like two images. People may also experience diplopia after receiving the COVID-19 vaccine, after contracting COVID-19, or following a COVID-19 infection. *Materials and Methods*: This review aims to summarize the cases of COVID-19 that can cause diplopia and its treatment in the past 5 years. The literature search databases used for this review were PubMed and Scopus. The keywords used were “diplopia,” “COVID-19,” and “treatment.” Sixteen articles were reviewed after screening and applying the inclusion criteria. *Results*: The results show that over the past 5 years, cases of diplopia related to COVID-19 have occurred in America, Europe, Asia, and Africa. Most studies are case reports, and the total number of patients was 26, with an age range of 14 to 81. *Conclusions*: The diplopia cases recovered within 1 day to 8 months. Patients who experienced diplopia after receiving the COVID-19 vaccine, during COVID-19 infection, or after COVID-19 infection exhibited different symptoms. Nasopharyngeal swabs, magnetic resonance imaging (MRI), computerized tomography (CT) scans, visual acuity tests, slit lamp biomicroscope examinations, eye movement tests, funduscopic examinations, and blood tests were the most commonly performed tests. Corticosteroids such as prednisone, methylprednisolone, and prednisolone were the most commonly used drugs to treat diplopia. In addition to corticosteroids, antibiotics, antivirals, antiplatelets, and vitamins were also given. An eye patch was considered to alleviate the diplopia.

## 1. Introduction

COVID-19 is a pandemic disease caused by Severe Acute Respiratory Syndrome Coronavirus 2 (SARS-CoV-2) that is assumed to have started as a zoonotic disease and spread quickly to people through respiratory droplets and contact [1]. The main symptoms of COVID-19 are dry cough, fever, dyspnea, and headache, with progression to pneumonia [2]. After infection with COVID-19, there are long-term effects on pulmonary, cardiovascular, neurological, hematological, multisystem inflammatory, renal, endocrine, gastrointestinal, and integumentary sequelae [3]. SARS-CoV-2 causes severe damage to tissues in other essential human organs such as the heart, kidney, liver, brain, and gastrointestinal system [4].

In the neurological system, the SARS-CoV-2 virus can influence nearly all neuro-ophthalmic pathways, impacting both afferent and efferent vision systems [5]. It is associated with numerous central nervous system disorders, including seizures, anosmia, posterior reversible encephalopathy syndrome (PRES), neuromyelitis optica (NMO) spectrum disorder, acute disseminated encephalomyelitis (ADEM), cerebral venous sinus thrombosis (CVST), cerebrovascular strokes, and peripheral nervous system conditions such as Guillain–Barré syndrome, Miller Fisher syndrome, polyneuritis cranialis, and myasthenia gravis [5]. SARS-CoV-2 exhibits ocular tropism, infecting the eyes directly or via migration through the trigeminal and optic nerves [6]. Like the blood–brain barrier, the blood–retinal barrier (BRB) maintains ocular immune privilege and metabolic homeostasis [6]. A study by Monu et al. in 2024 provided the first evidence that SARS-CoV-2 can infect retinal cells via the BRB, leading to retinal inflammation [7]. In addition, the disease’s effects may manifest and cause eye complications. The most common eye symptoms include blurred vision, conjunctival secretions, dry eyes, foreign body sensation, itching, eye pain, photophobia, and tearing [8]. Another symptom is diplopia. 

Diplopia, or double vision, is a medical condition that makes one object appear like two images [9]. The two images may have the same brightness, or one may appear shadowy or weak [10]. Diplopia can be either monocular or binocular, however, diplopia in COVID-19 is binocular due to ophthalmoplegia. Ophthalmoplegia is defined as the paralysis or weakness of the eye muscles, which causes diplopia, fuzzy vision, and/or squinting. The disorder is commonly divided into two types: chronic progressive external ophthalmoplegia and internal ophthalmoplegia [11]. COVID-19 can affect any cranial nerve; however, the most commonly affected are VII, VI, and III [12]. Binocular diplopia normally happens when both eyes are open and disappears when one eye is closed, but monocular diplopia continues even when one eye is closed [10]. Several studies have reported that diplopia may occur in people infected with COVID-19 or after receiving the COVID-19 vaccine [13,14]. In addition, diplopia can also occur in people after COVID-19 infection. Kang et al. reported cases of binocular diplopia in post-vaccine patients [15]. Manolopoulos et al. reported a case complaining of diplopia caused by SARS-CoV-2 infection [16]. Bista et al. reported a case of diplopia where the patient had a history of COVID-19 infection [17].

A review article in 2022 by Siddiqi et al. in Pakistan, reported that COVID-19 can cause Miller–Fisher syndrome [18]. Another review article in 2023 by Khor et al. in Malaysia reported that COVID-19 can cause strabismus [19]. However, since several case studies of COVID-19 causing diplopia have been reported in many countries, no review article summarizes how COVID-19 can cause diplopia or gives insights into the incidence, clinical presentation patterns, diagnostic considerations, and treatment outcomes. Therefore, we narratively summarize the cases of COVID-19 that caused diplopia and how they were treated in the past five years.

## 2. Methods

### 2.1. Data Sources and Search Strategy

A literature search was conducted using the PubMed and Scopus databases in May 2024. The keywords used were “diplopia,” “COVID-19,” and “treatment.” The search details are ((diplopia) AND (COVID-19)) AND (treatment). The criteria for a report to be included in this review were articles that reported diplopia and COVID-19, English-language articles, treatment was given to overcome diplopia, and the publication date was within the past 5 years. We excluded preprints, non-peer-reviewed articles, and articles with diplopia resulting from other virally induced conditions. We applied the PRISMA flow chart to illustrate the article selection process [20].

### 2.2. Data Extraction

This review carefully extracts key data points, such as information relating to authors, years, country, type of study, number of patients, age, time when diplopia occurred, subjective data, examination, examination results, diagnosis, treatment and dose, duration of treatment, and results.

## 3. Results

**Figure 1:**
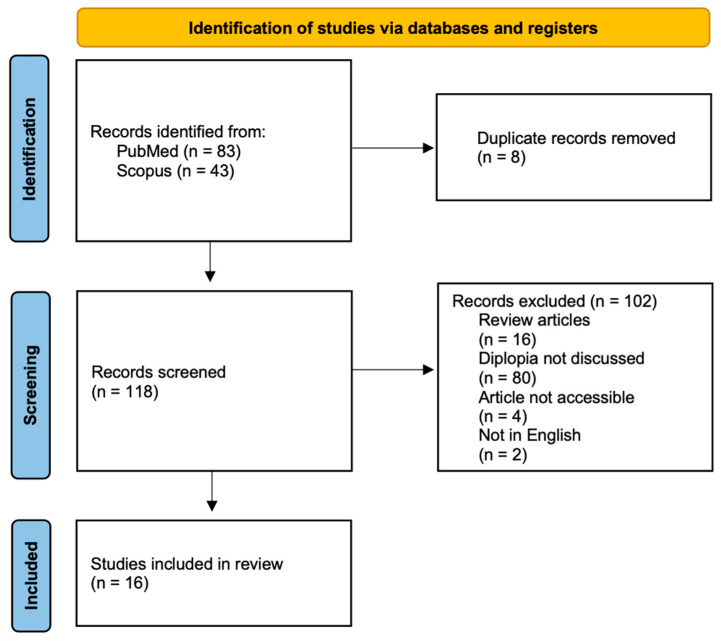
PRISMA flowchart for the literature search.

Figure 1 illustrates the PRISMA flow chart for the article selection process. The initial search yielded 126 articles: 83 from PubMed and 43 from the Scopus database. Following the initial search, duplicate files were removed until 118 articles were obtained. The second round of selection eliminated 102 articles. As a result, 16 studies were included in the review process. 

Table 1 displays the list of articles included in this study. The publications covered the period from 2020 until 2023. The cases occurred in the Americas, such as the USA, Canada, and Brazil; European countries, such as Greece, Italy, Croatia, and France; Asian countries, such as Nepal, Korea, Taiwan, Japan, India; and Morocco in Africa. In terms of article type, there were 13 standard case reports, 1 case report plus literature review, 1 case series, and 1 retrospective study. A total of 26 patients with an age range of 14–81 reported diplopia related to COVID-19. Out of thirty patients reported, 17 patients had diplopia due to COVID-19 vaccination, 7 patients had diplopia due to COVID-19 infection, and 2 patients had diplopia due to a history of COVID-19 infection. Of the 17 people affected by diplopia due to the COVID-19 vaccine, 7 patients received the vaccine from Pfizer, 5 patients received the vaccine from Astra Zeneca, 1 patient received vaccines from both Astra Zeneca and Pfizer, and 4 patients received the vaccine from Moderna.

Table 2 presents a summary of articles related to diplopia and COVID-19. The summary includes the time when diplopia occurred, subjective data, examination results, diagnosis, treatment, duration of treatment/observation, and the outcomes.

All patients experienced diplopia following COVID-19 vaccination or infection. The symptoms appeared from 1 day to several weeks after COVID-19 vaccination or infection. Most patients reported additional signs such as blurred vision, eye movement pain, or periorbital swelling. Ophthalmologists conducted examinations of the eye and nerve, performed imaging (MRI, CT), and ordered lab tests to make the diagnosis. The results revealed various findings such as cranial nerve palsies, ocular myasthenia gravis, and, in some cases, autoimmune markers or inflammatory changes. Treatments varied depending on the underlying cause and included corticosteroids, pyridostigmine, intravenous immunoglobulin, antivirals, and supportive therapies like eye patches. Recovery durations ranged from 1 day to 8 months.

## 4. Discussion

### 4.1. Diplopia Symptoms Related to COVID-19

A total of seventeen patients experienced acute-onset binocular diplopia shortly after receiving the COVID-19 vaccine. These patients often report additional ocular symptoms, such as periorbital swelling, pain when moving the eye, and blurred vision [14]. The onset of symptoms varied from between 1 and 4 days after vaccination [26].

The other seven patients described binocular diplopia along with a confirmed COVID-19 infection. These cases exhibit a wider range of symptoms beyond ocular complaints, including respiratory symptoms, headaches, and malaise [28]. In these patients, the duration of diplopia was generally longer, with some cases lasting up to 10 days [30].

Two other patients that were previously infected with COVID-19 experienced diplopia. These two cases had other ophthalmic symptoms, including decreased visual acuity, periorbital inflammation, and eyelid ptosis. Most importantly, some of these patients had a history of pre-existing eye conditions or autoimmune disorders [17,31].

COVID-19 has been associated with a variety of neuro-ophthalmologic symptoms, the majority of which are connected to demyelinating illness. While the mechanism of these manifestations is uncertain, ideas include direct neural invasion, endothelial cell failure leading to ischemia and coagulopathy, or a widespread inflammatory “cytokine storm” caused by the virus [32].

The virus is not the only respiratory virus that can cause neurological issues, as influenza, TB, and SARS-CoV-1 can [33]. SARS-Cov-2 is not primarily neurotropic, as only a small percentage of autopsies reveal viral presence in the brain [33]. As a result, neurological problems such as an abnormal immune response are thought to be caused by indirect viral impacts [33]. This could include increased cytokine levels, blood–brain barrier disruption, immunological infiltration, blood vessel inflammation, and blood vessel blockage resulting in hypoxia, as well as immune-mediated tissue damage caused by cells and/or autoantibodies [33].

### 4.2. Examination

To confirm the patient’s illness, several tests were performed. A common laboratory test (8 out of 16 articles) was using reverse transcription polymerase chain reaction (RT-PCR) to detect SARS-CoV-2 from nasopharyngeal swabs to determine whether the patient was infected or not [13,16,25,27,28,29,30,31]. In addition to the nasopharyngeal swab, the most commonly used examination was MRI (magnetic resonance imaging), which was used in 8 articles out of the 16 articles obtained [13,15,17,24,25,26,27,28]. A computed tomography (CT) scan is also a commonly used examination, with 3 articles out of the 16 articles obtained using this technique [16,21,29]. In the other four articles, a combination of both MRI and CT scans was used [14,22,23,31].

The majority of cases tested using MRI and/or CT scans reported unremarkable results. Reshef et al. reported that the examination showed inflammation of the left superior oblique muscle in patient 1, inflammation and enlargement of the left medial and lateral rectus in patient 2, and inflammation with enlargement of the left lacrimal gland in patient 3 [14]. Kang et al. reported that the examination showed right distal internal carotid artery aneurysm in patient 6; mild to moderate small vessel disease in the cerebral white matter in patient 7; a small acute infarct with restricted diffusion in patient 8; and left eyelid edema with contrast enhancement in patient 10 [15]. Signa et al. reported that the examination showed hyperintensity of the mesencephalic tegmentum and periaqueductal region [27].

Kang et al. also reported that ten patients underwent a Hess screen test [15]. The Hess screen test is a method to accurately measure eye alignment in three dimensions [15]. The Hess screen test findings revealed palsies of the left and right fourth cranial nerves, as well as palsies of the third and sixth cranial nerves in the left eye and the sixth cranial nerve in the right eye [15].

Besides diplopia examination, there are eight articles that examine the visual acuity of the patients. Reshef et al. reported that the results of visual acuity showed normal vision at 20/20 in both eyes, 20/15 in both eyes, and uneven vision with 20/20 in the right eye and 20/40 in the left eye, suggesting a significant difference in visual clarity between the two eyes [14]. Choi et al. reported that the visual acuity was 20/40 in the right eye and 20/20 in the left eye [24]. Pappaterra et al. reported that the results of the visual acuity test showed 20/40 in the right eye and 20/30 +2 in the left eye [26]. Zayet et al. reported that visual acuity tests showed normal vision at 20/20 in both eyes, 20/20 in both eyes, and uneven vision with 20/63 in the right eye and 20/16 in the left eye [28]. Medeiros et al. reported visual acuity results showing normal vision at 20/20 in both eyes [29]. Vasanthpuram et al. reported that the results of visual acuity were 6/6p and 6/12 in the right and left eye, respectively [30]. Bista et al. reported that the result of visual acuity was 20/20 in the oculus uterque [17]. Additionally, an intraocular pressure examination was performed, and the results were normal [14,15]. These visual acuity results are not affected by COVID-19 occurring alongside diplopia, as ophthalmoplegia mainly involves cranial nerves III, IV, and VI, which are responsible for extraocular muscle function [12]. These nerves are different from cranial nerve II (optic nerve), which is responsible for visual acuity [12].

Laboratory blood examinations were also performed, including CBC, BMP, ESR, CRP, ACE, lysozyme, IgG subclass, ANA, ANCA, DS-DNA, TSH with reflex, SS-A/SS-B, RF, quantiferon-gold, FTA-ABS, RPR, EBV, Lyme screen with reflex, thyroxine, complement C3, complement C4, and anti-AChR antibody [14]. In these blood examinations, all cases reported unremarkable results [14,21,23,25]. Besides that, laboratory blood examinations for antibodies IgM and IgG can also reveal COVID-19 infections that cannot be detected by nasopharyngeal swabs (throat swab PCR and CSF-PCR) [27]. Following all laboratory blood tests, ophthalmologic examination including anterior and posterior segment parameters, neurologic examination, funduscopic examination, physical examination, clinical examination, sensorimotor evaluation, and antibody tests, all examination results were mostly normal [24,28,30]

Moreover, many additional examinations were also performed, such as the sustained upward test, single-fiber electromyography (SFEMG) test, and repetitive stimulation test. The result of the ophthalmological examination was that the left eye could not look up; the result of the sustained upward test was the presence of a movement like “reverse ocular bobbing” in the left eye; and the result of the SFEMG test was the presence of abnormal jitter in 5 of the 18 muscle fiber pairs sampled [21]. Slit lamp tests were also performed in three cases, and the results were normal in two cases and showed mild superficial punctate keratitis with dendritic-patterned lesions in one case [13,17,26]. Apart from the slit lamp test, it is also necessary to perform a worth-four-dot test (WFDT) and diplopia charting to diagnose diplopia. The diplopia chart showed non-crossing diplopia [17]

### 4.3. Treatment

Most patients commonly receive corticosteroids in the treatment of diplopia, such as prednisone, methylprednisolone, prednisolone, and dexamethasone. Reshef et al. reported that the dose of prednisone used was 60 mg for 1 month [14]. While Abicic et al. reported the dose of prednisone used was 10 mg daily, in the following weeks it was gradually raised to 20 mg daily for 2 months [23]. For methylprednisolone, the oral dose is 250 mg once a day, while the dose of methylprednisolone is pulsed at 30 mg/kg/d for 3 days, with a subsequent shift to oral prednisone at 1 mg/kg/d on the fourth day [15,27]. The dose of prednisolone given in Su et al.’s report was 40 mg daily for 3 weeks and in Choi et al.’s report was 1 g/day for five days with tapering [21,24]. Bista et al. reported an oral corticosteroid dose of 60 mg tapered weekly for 2 months [17]. While for dexamethasone, the dose used was 8 mg for 7 days, with a taper over 5 days of 6 mg TID, 4 mg TID, 2 mg TID, 2 mg BID, and 2 mg OD then stop [31].

Corticosteroids are commonly used and appear to be effective in treating diplopia associated with COVID-19. Several studies have reported the effectiveness of corticosteroids in ophthalmoplegia affected by COVID-19. For instance, Iwasaki et al. observed that while some cases of third cranial nerve palsy due to COVID-19 infection were treated with antiviral medications, methylprednisolone, and immunoglobulin, others recovered with only supportive care, indicating the potential for spontaneous improvement [34]. Similarly, Tan et al. reported that isolated oculomotor nerve palsy associated with COVID-19 has an excellent prognosis, with often complete and early recovery, with or without short-term oral steroids [35]. Corticosteroids were more commonly administered due to their well-established anti-inflammatory properties, particularly in cases with suspected immune-mediated complications.

Infected patients received several antibiotics and antivirals in addition to corticosteroids. The antibiotics given were azithromycin and doxycycline. The first day’s dosage of azithromycin was 500 mg, followed by 250 mg daily for 6 days in accordance with the Moroccan national guidelines for the management of COVID-19 in adults [13,29]. Meanwhile, doxycycline was given two times per day as off-label treatment started by a general physician [30]. During the early stages of the COVID-19 pandemic, antibiotics were widely prescribed and were likely to address potential or suspected secondary bacterial infections rather than for their direct antiviral or immunomodulatory effects [36]. Antivirals such as valacyclovir and acyclovir were also given to the patients in Nanatsue, K. et al.’s study, considering the possibility of Bell’s palsy [25], and Bista, B. et al.’s [17] report.

In addition, patients received pyridostigmine at 180 mg daily and, in the second week, this was increased to 300 mg daily for 2 months [23]. Antiplatelet therapy (aspirin) was initiated for patients with ocular motor nerve palsy due to likely vascular causes in Kang’s report [15]. Topical anti-viral ointment (Ocuvir 3% *w*/*w*) was also given five times for two weeks in Bista, B. et al.’s [17] report.

Vitamins are also given to patients with diplopia who are infected with COVID-19. Nanatsue et al. reported that mecobalamin was given for 1 month [25]. In addition, vitamin C (1 g twice a day) and zinc (90 mg twice a day) were given in Belghmaidi’s report [13]. Vasanthapuram et al. also reported vitamin B12 supplementation once a day and vitamin C supplementation for 10 days [30].

Apart from using oral and topical medications, there are also eye patches that can be used to treat diplopia. Zayet et al. reported that the only medication taken by the patient was an eye patch prescribed for the patient’s comfort and quality of life for 2 months [28]. Additionally, Tremblay and Medeiros et al. reported that the patient used an eye patch in addition to oral medication [29,31]. The diplopia cases recovered within 10 days to 8 months. Recurrences of COVID-19-associated diplopia appear to be uncommon. Notably, Zayet at al. reported that the patient’s diplopia resolved completely following a normal ophthalmological exam, with no evidence of relapse [28].

### 4.4. Future Recommendation

The 16 articles included in this paper have numerous patient examinations. To be more efficient, the doctors might carefully select which examinations are necessary. In addition to the number of examinations, the medications given to patients with side effects also need to be considered. For example, corticosteroids are one of the primary treatments for diplopia. However, long-term corticosteroid use may cause serious side effects; one of them is bone loss [37]. Calcium and vitamin D supplements may be required to prevent and treat corticosteroid-induced bone loss [37]. Another suggestion, according to Medeiros, is a more conservative approach with orthoptic therapy, ocular patching, and Fresnel prisms before considering more invasive therapies such as botulinum toxin or other surgical approaches [29].

## 5. Conclusions

The COVID-19-related diplopia cases were reported in four continents, including America, Europe, Asia, and Africa. The total number of patients was 26, with an age range of 14 to 81. Patients who experience diplopia after receiving the COVID-19 vaccine, while currently infected with COVID-19, or when they have a history of COVID-19 infection have different symptoms. The most commonly performed tests are nasopharyngeal swabs, MRIs, CT scans, visual acuity tests, and blood tests. Corticosteroids like prednisone, methylprednisolone, and prednisolone are the most commonly used in the treatment of diplopia. In addition to corticosteroids, doctors also administer antibiotics, antivirals, vitamins, antiplatelets, and eye patches. All patients recovered within 1 day to 8 months. Additional supplements, such as calcium and vitamin D, may be required by patients on long-term corticosteroid therapy to counteract its side effects.

## Figures and Tables

**Table 1:** List of articles related to diplopia and COVID-19.

Article Number	Authors	Years	Country	Type of Study	Number of Patients	Age	Time When Diplopia Occurred
1	Reshef, E. R. et al. [14]	2022	USA	Case report	2	68	After receiving the COVID-19 vaccine (Pfizer/BioNTech)
						33	After receiving a second dose of the COVID-19 vaccine (Moderna)
2	Kang, K. et al. [15]	2023	Korea	Retrospective study	9	56–81	After receiving the COVID-19 vaccination (Pfizer 4 patients, Astra Zeneca 4 patients, both Astra Zeneca and Pfizer 1 patient)
3	Su, W. Y. et al. [21]	2023	Taiwan	Case report	1	39	After receiving the first dose of the Pfizer-BioNTech COVID-19 vaccine
4	Hoshina, Y. et al. [22]	2022	Japan	Case report	1	30	After receiving the Moderna vaccination
5	Abicic, A. et al. [23]	2022	Croatia	Case report	1	65	After receiving the Pfizer-BioNTech vaccine
6	Choi, S. Y. et al. [24]	2023	Korea	Case report	1	71	After receiving the COVID-19 Astra Zeneca vaccine
7	Nanatsue, K. et al. [25]	2022	Japan	Case report	1	72	After receiving the Moderna vaccine
8	Pappaterra, M. C. et al. [26]	2023	USA	Case report	1	81	After receiving the first dose of the COVID-19 vaccine (Moderna)
9	Belghmaidi, S. et al. [13]	2020	Morocco	Case report	1	24	Infected with COVID-19
10	Manolopoulos, A. et al. [16]	2022	Greece	Case report and literature review	1	41	Infected with COVID-19
11	Signa, S. et al. [27]	2022	Italy	Case report	1	14	Infected with COVID-19
12	Zayet, S. et al. [28]	2023	France	Case series	2	56–63	Infected with COVID-19
13	Medeiros, A. L. et al. [29]	2022	Brazil	Case report	1	48	Infected with COVID-19
14	Vasanthpuram, V. H. et al. [30]	2021	India	Case report	1	58	Infected with COVID-19
15	Bista, B. et al. [17]	2023	Nepal	Case report	1	39	Post-infection with COVID-19
16	Tremblay, C. et al. [31]	2023	Canada	Case report	1	40	Post-infection with COVID-19
				Total patients = 26			

**Table 2:** Summary of articles related to diplopia and COVID-19.

Article Number	Time When Diplopia Occurred	Subjective Data	Examination	Examination Results	Diagnosis	Treatment	Duration of Treatment /Observation	Outcomes
1	After receiving the COVID-19 vaccine (Pfizer/BioNTech)	Patient 1: Unknown autoimmune history presented with binocular diplopia, pain with extraocular movements, and periorbital swelling 4 days after receiving the second dose of the COVID-19 vaccine.	Visual acuity Intraocular pressures (IOP) Orbital CT/MRI Laboratory test: CBC, BMP, ESR, CRP, ACE, lysozyme, IgG subclass, ANA, ANCA, DS-DNA, TSH with reflex, SS-A/SS-B, RF, QuantiFERON-gold, FTA-ABS, RPR, EBV, Lyme screen with reflex, thyroxine, complement C3, and complement C4	Visual acuity: 20/20 in each eye; 20/15 in each eye; 20/20 in the right eye; and 20/40 in the left eye IOP: normal Orbital CT/MRI: inflammation of the left superior oblique muscle; inflammation and enlargement of the left medial and lateral rectus; inflammation with left lacrimal gland enlargement Laboratory test: All the tests were unremarkable	Binocular diplopia	60 mg of oral prednisone	1 month	All patients achieved a complete resolution.
	After receiving a second dose of the COVID-19 vaccine (Moderna)	Patient 2: Binocular diplopia, periorbital swelling, and pain with extraocular movements 1 day following the second dose of the COVID-19 vaccine.			Binocular diplopia			
2	After receiving the COVID-19 vaccination	9 patients with diplopia after the COVID-19 vaccination.	Best-corrected visual acuity Intraocular pressure The Hess screen test Brain MRI	Best-corrected visual acuity: 1.0; 1.0; 0.63 and 0.5; 0.8 and 0.6; 0.5 and 0.32; 0.8; 1.0; 1.0 and 0.8; 1.0. Intraocular pressure: 14 mmHg; 21 and 19 mmHg; 12 and 10 mmHg; 21 and 22 mmHg; 13 mmHg; 13 mmHg; 11 and 12 mmHg; 16 and 15 mmHg; 17 mmHg; 31 mmHg The Hess screen test: left third nerve palsy; left fourth nerve palsy; left sixth nerve palsy; right sixth nerve palsy; right fourth nerve palsy; left fourth nerve palsy; right fourth nerve palsy; left fourth nerve palsy; normal; Brain MRI: no specific findings; unremarkable; not mentioned; unremarkable; unremarkable; right distal internal carotid artery aneurysm; mild to moderate small vessel disease in the cerebral white matter; a small acute infarct with restricted diffusion; unremarkable; left eyelid edema with contrast enhancement	8 patients had nerve palsy with diplopia; 1 patient diplopia (subjective)	Antiplatelet therapy was initiated for three of eight patients with ocular motor nerve palsy (cases 1, 7, and 8) due to likely vascular causes. One patient (case 6) continued clopidogrel without additional treatment	1 to 5 months observation	Diplopia resolved varies between 1 day to 6 months.
3	After receiving the first dose of the Pfizer-BioNTech COVID-19 vaccine	The patient noticed diplopia 1 week after accepting the first dose of the COVID-19 vaccine. The patient had transient blurred vision when looking toward the left side.	Blood test Ophthalmological examination Sustained upward test Single-fiber electromyography (SFEMG) test Anti-AChR antibodies Repetitive stimulation test CT scan	Blood test: had anti-thyroglobulin and anti-SSA antibodies Ophthalmological examination: the left eye was unable to move upwards Sustained upward test: movement like “reverse ocular bobbing” of the left eye SFEMG test: an abnormal jitter in 5 of 18 muscle fiber pairs sampled Anti-AChR antibody: negative Repetitive stimulation test: normal CT scan: no mediastinal mass	Ocular myasthenia gravis with diplopia	Prednisolone, 40 mg every day	3 weeks	Fully recovered, except for transient diplopia lasting about 5–10 min.
4	After receiving the Moderna vaccination	Patient developed acute onset diplopia, 2 days after receiving the first dose of Covid-19 vaccination. Patient complained of blurred vision with horizontally displaced images, which worsened with increased eye strain.	Cranial nerves, sensory examination and deep tendon reflexes AChR antibody MuSK antibody Chest CT and MRI	Cranial nerves, sensory examination, and deep tendon reflexes: normal AChR antibody: borderline high MuSK antibody: negative Chest CT and MRI: unremarkable	Myasthenia gravis with diplopia	Oral pyridostigmine, 30 mg three times a day Prednisone, 10 mg a day	Not mentioned	The symptoms improved but continued to fluctuate.
5	After receiving the Pfizer-BioNTech vaccine	The patient’s complaint of vertical diplopia was present continuously, regardless of the time of day, with no signs or symptoms of other muscle weakness after 3 weeks of receiving the booster dose of the COVID-19 vaccine.	Neurological examination CT scan of the brain Routine laboratory examinations Brain MRI Intramuscular prostigmin test Chest CT scan Repetitive nerve stimulation test	Neurological examination: normal CT scan of the brain: unremarkable Routine laboratory examination: normal Brain MRI: small nonspecific subcortical white matter T2 hyperintense lesions Intramuscular prostigmin test: negative Chest CT scan: no sign of thymoma Repetitive nerve stimulation test: negative	Diplopia in all directions, except when looking up and straight	Pyridostigmine 180 mg daily, in the second week, was raised to 300 mg daily Added 10 mg of prednisone daily. In the following weeks, the dose of prednisone was gradually raised to 20 mg daily	2 months	Vertical diplopia is only in the far left and down eye positions.
6	After receiving the COVID-19 Astra Zeneca vaccine	The patient presented with an acute onset of painless diplopia and visual disturbance for 2 days and received the first COVID-19 vaccine.	Visual acuity Fundoscopy examination Routine laboratory examinations Antinuclear, anti-Smith, anti-cardiolipin, anti-dsDNA IgG antibodies, lupus anticoagulant Anti-dsDNA IgM CSF study An orbital MRI and brain MRA	Visual acuity: 20/40 in the right eye and 20/20 in the left eye Fundoscopy examination: normal bilaterally Routine laboratory examination: normal Antinuclear, anti-Smith, anti-cardiolipin, anti-dsDNA IgG antibodies, lupus anticoagulant: negative Anti-dsDNA IgM: positive CSF study: unremarkable An orbital MRI and brain MRA: hyperintense lesion with enhanced protruding in the right cavernous sinus	Diplopia	Prednisolone 1 g/day for five days continue with tapering	1 month	Fully recovered.
7	After receiving the Moderna vaccine	The patient complained of diplopia that manifested 1 week after receiving the second dose of the COVID-19 vaccine.	Neurological examination Blood test Nasopharyngeal swab Head MRI and MRA	Neurological examination: revealed mild ocular motor restriction in all directions Blood test: normal, except for prolongation of the prothrombin time Nasopharyngeal swab: negative The head MRI and MRA show no obvious abnormalities.	Miller–Fisher syndrome with diplopia	Intravenous immunoglobulin Steroids Valacyclovir Mecobalamin	22 days	Symptoms gradually improved
8	After receiving the first dose of the COVID-19 vaccine (Moderna)	The patient complained of binocular oblique diplopia after receiving the first dose of the COVID-19 vaccine four days ago.	Visual acuity Sensorimotor evaluation Slit lamp examination Brain MRI scan and MRA Erythrocyte sedimentation rate and elevated C-reactive protein level	Visual acuity: 20/40 in the right eye and 20/30 +2 in the left eye Sensorimotor evaluation: exotropia of 3 prism diopters in the primary position Slit lamp examination: normal bilaterally Brain MRI scan and MRA: no pathology to explain the patient’s complaints Erythrocyte sedimentation rate and elevated C-reactive protein level: normal	Binocular diplopia	Not mentioned	Not mentioned	The patient had full extraocular motility in both eyes and minimal residual exodeviation in the primary position, indicating a spontaneous near-total resolution of his cranial neuropathy.
9	Infected with COVID-19	Acute onset of diplopia and strabismus of the left eye that occurred 3 days later.	Ophthalmic and neurological examinations Slit lamp and fundus examinations Nasopharyngeal swab Oculo-cerebral MRA Blood test	Ophthalmic and neurological examination: visual acuity 0.1 logMAR in both eyes, acute painless incomplete palsy of the third cranial nerve was suspected Slit lamp and fundus examinations: unremarkable Nasopharyngeal swab: positive Oculo-cerebral MRA: unremarkable Blood test: mild normocytic regenerative anemia	Diplopia	Chloroquine 500 mg, 2 times a day Azithromycin 500 mg once a day the first day, then 250 mg every day for 6 days, Vitamin C: 1 g, 2 times a day Zinc 90 mg, 2 times a day	10 days	Complete recovery.
10	Infected with COVID-19	The patient complained of headaches, unresolved pain with common analgesics, and double vision over the last 2 days with an unknown medical history.	Physical examination Ophthalmology consultation CT scan and chest x-ray Routine blood tests Nasopharyngeal swab	Physical examination: revealed a limitation to the abduction of the right eye due to palsy of the right lateral rectus muscle causing painless, binocular, horizontal diplopia in the right gaze Ophthalmology consultation: normal CT scan and chest x-ray: no evidence of acute pathology was observed Routine blood tests: low white blood cell count along with lymphopenia Nasopharyngeal swab: positive	Diplopia	No medical treatment except for acetaminophen for headache relief	Not mentioned	Diplopia had been completely resolved after 1 month.
11	Infected with COVID-19	The patient presented with a 2-day history of binocular diplopia, particularly evident for distant vision, without any other neurologic symptoms.	Nasopharyngeal swab Physical examination Fundus examination Brain MRI Spinal MRI Routine blood test SARS-CoV-2 serology CSF analysis	Nasopharyngeal swab: negative Physical examination: normal Fundus examination: normal Brain MRI: hyperintensity of the mesencephalic tegmentum and periaqueductal region Spinal MRI: normal Routine blood test: normal SARS-CoV-2 serology: positive CSF analysis: increased glycorrhachia and pleocytosis	Binocular diplopia	Methylprednisolone pulses 30 mg/kg/d for 3 days with a subsequent shift to oral prednisone 1 mg/kg/d on the fourth day with a course of intravenous immunoglobulin 400 mg/kg/d for 5 days Steroid therapy was progressively tapered and finally stopped after further 2 months	4 months	Gradual improvement of nystagmus and diplopia with complete resolution in a few days was observed and at the 4-month follow up, neurologic examination and neuroimaging were normal.
12	Infected with COVID-19	Patient 1: complained of binocular diplopia, concomitant to frontal headaches and new loss of taste. Patient 2: admitted for dyspnea and diplopia.	All patients: Visual acuity Anterior and posterior segment parameters Respiratory and neurologic examinations Brain MRI Nasopharyngeal swab Patient 2 had additional Clinical examination	All patients Respiratory and neurologic examinations: all patients normal, expect diplopia Nasopharyngeal swab: positive; positive. Respiratory and neurologic examinations: all patients normal, expect diplopia Brain MRI: normal Nasopharyngeal swab: positive; positive. Patient 2: Clinical examination: proptosis of the right eye with a contralateral lagophthalmos	Patient 1: Binocular diplopia Patient 2: Diplopia	Patient 1 and 2 only prescribed eye patches for the patient’s convenience and life quality.	1. 2 months 2. 1 month	Diplopia resolved.
13	Infected with COVID-19	The patient complained of acute diplopia and clinical manifestations of the SARS-CoV-2 infection. One day prior to presenting diplopia, the patient took a pill of cyclobenzaprine hydrochloride 10 mg.	Chest CT Nasopharyngeal swab Visual acuity Abduction limitation Ophthalmologic examinations	Chest CT: normal Nasopharyngeal swab: positive Visual acuity: 20/20 in both eyes Abduction limitation: was observed in the left eye Ophthalmologic examinations: normal	The sixth cranial nerve palsy with acute diplopia	Oral azithromycin and ivermectin for COVID-19 treatment. Eye patch a few hours each day	8 months	The diplopia recovered eight months later.
14	Infected with COVID-19	Patient complained of binocular diplopia for 10 days and infected with COVID-19.	Nasopharyngeal swab Examination Visual acuity Color vision Anterior segment Posterior segment Blood test	Nasopharyngeal swab: positive Examination: vertical diplopia, enhanced in downgaze and levoversion Visual acuity: 6/6p and 6/12 in the right and left eyes Color vision: normal Anterior segment: bilateral nasal pterygium and posterior polar cataract in the left eye Posterior segment: unremarkable Blood test: suggested for diabetes mellitus	Binocular diplopia	Vitamin B12 supplements once daily Oral doxycycline two times per day Ivermectin once daily Vitamin C supplementation for 10 days. Metformin 500 mg once daily	1 month	The diplopia was relieved.
15	Post-infection with COVID-19	The patient complained of binocular diplopia and did not have any comorbidities.	Visual acuity, Slit lamp test, The worth-four-dot test (WFDT), Diplopia charting, MRI	Visual acuity: 20/20 in the oculus uterque Slit lamp test: mild superficial punctate keratitis with a dendritic patterned lesion WFDT: the eye movements were restricted to the left eye’s lateral gaze and showed diplopia Diplopia charting: uncrossed diplopia MRI: did not show any significant pathological changes	Binocular diplopia	Topical antiviral ointment (Ocuvir 3% *w*/*w*) 5 times for two weeks Oral corticosteroid 60 mg, tapered weekly Systemic antiviral therapy (Acyclovir 400 mg) twice daily	2 months	The diplopia resolved, and the WFDT showed fusion.
16	Post-infection with COVID-19	The patient complained of moderate diplopia, was unable to focus with binocular vision, and developed eyelid ptosis two days later. Patient previously infected with the Omicron variant of COVID-19.	CT head, CT angiogram, and MRI Nasopharyngeal swab	CT head, CT angiogram, and MRI: no intracranial or vascular pathology was identified Nasopharyngeal swab: negative	Diplopia	Treatment with a left eye patch Dexamethasone 8 mg PO TID for 7 days, with a taper over 5 days of 6 mg TID, 4 mg TID, 2 mg TID, 2 mg BID, and 2 mg OD then stop	52 days	Diplopia was no longer detected.

Notes: CBC, complete blood count; BMP, basic metabolic panel; ESR. erythrocyte sedimentation rate; CRP, C-reactive protein; ACE, angiotensin-converting enzyme; ANA, antinuclear antibody; ANCA, antineutrophil cytoplasmic antibody; DS-DNA, double-stranded DNA antibody; TSH, thyroid-stimulating hormone; SS-A/SS-B, Sjogren’s antibodies; RF, rheumatoid factor; FTA-ABS, fluorescent treponemal antibody-absorption; RPR, rapid plasma reagin; EBV, Epstein–Barr virus antibodies; AChR, the acetylcholine receptor; MuSK, muscle-specific tyrosine kinase; CT, computed tomography; MRI, magnetic resonance image; MRA, magnetic resonance angiography; PO, per oral; TID, ter in die, three times a day; BID, bis in die, twice a day; OD; once daily.

## Abbreviations

The following abbreviations are used in this manuscript:

CBC	complete blood count
BMP	basic metabolic panel
ESR	erythrocyte sedimentation rate
CRP	C-reactive protein
ACE	angiotensin-converting enzyme
ANA	antinuclear antibody
ANCA	antineutrophil cytoplasmic antibody
DS-DNA	double-stranded DNA antibody
TSH	thyroid-stimulating hormone
SS-A/SS-B	Sjogren’s antibodies
RF	rheumatoid factor
FTA-ABS	fluorescent treponemal antibody-absorption
RPR	rapid plasma reagin
EBV	Epstein–Barr virus antibodies
AChR	acetylcholine receptor
MuSK	muscle-specific tyrosine kinase
CT	computed tomography
MRI	magnetic resonance image
MRA	magnetic resonance angiography
PO	per oral
TID	ter in die, three times a day
BID	bis in die, twice a day
OD	once daily.

## References

[1] Lu R., Zhao X., Li J., Niu P., Yang B., Wu H., Wang W., Song H., Huang B., Zhu N. (2020). Genomic Characterisation and Epidemiology of 2019 Novel Coronavirus: Implications for Virus Origins and Receptor Binding. Lancet.

[2] Zhou P., Yang X.L., Wang X.G., Hu B., Zhang L., Zhang W., Si H.R., Zhu Y., Li B., Huang C.L. (2020). A Pneumonia Outbreak Associated with a New Coronavirus of Probable Bat Origin. Nature.

[3] Joshee S., Vatti N., Chang C. (2022). Long-Term Effects of COVID-19. Mayo Clinic Proceedings.

[4] Shah M.D., Sumeh A.S., Sheraz M., Kavitha M.S., Venmathi Maran B.A., Rodrigues K.F. (2021). A Mini-Review on the Impact of COVID-19 on Vital Organs. Biomed. Pharmacother..

[5] Feizi M., Isen D.R., Tavakoli M. (2023). Neuro-Ophthalmic Manifestations of Coronavirus Disease 2019 and Its Vaccination: A Narrative Review. J. Ophthalmic Vis. Res..

[6] Zhao Y., Tang Y., Wang Q.Y., Li J. (2025). Ocular Neuroinflammatory Response Secondary to SARS-CoV-2 Infection-a Review. Front. Immunol..

[7] Monu M., Ahmad F., Olson R.M., Balendiran V., Singh P.K. (2024). SARS-CoV-2 Infects Cells Lining the Bloodretinal Barrier and Induces a Hyperinflammatory Immune Response in the Retina via Systemic Exposure. PLoS Pathog..

[8] Akbari M., Dourandeesh M. (2022). Update on overview of ocular manifestations of COVID-19. Front. Med..

[9] Ungureanu L., Irincu L., Diaconu S., Oprițoiu B., Chaudhuri K.R., Falup-Pecurariu C. (2024). Diplopia in Movement Disorders: A Systematic Review of the Literature. J. Pers. Med..

[10] Jain S. (2022). Diplopia: Diagnosis and Management. Clin. Med. J. R. Coll. Physicians Lond..

[11] Gomes P.F., Momen A.B.I., Sultana A., Alam R.F., Saber S., Alam M.T. (2021). COVID-19 Presenting with Ophthalmoplegia in a Patient with Acute Kidney Injury. Bangladesh Crit. Care J..

[12] Finsterer J., Scorza F.A., Scorza C.A., Fiorini A.C. (2022). COVID-19 Associated Cranial Nerve Neuropathy: A Systematic Review. Bosn. J. Basic Med. Sci..

[13] Belghmaidi S., Nassih H., Boutgayout S., Fakiri K.E., Qadiri R.E., Hajji I., Bourahouate A., Moutaouakil A. (2020). Third Cranial Nerve Palsy Presenting with Unilateral Diplopia and Strabismus in a 24-Year-Old Woman with COVID-19. Am. J. Case Rep..

[14] Reshef E.R., Freitag S.K., Lee N.G. (2022). Orbital Inflammation Following COVID-19 Vaccination. Ophthalmic Plastic and Reconstructive Surgery.

[15] Kang K., Lee S.Y., Lee D.C. (2023). Neuro-Ophthalmologic Symptoms after Coronavirus Disease 2019 Vaccination: A Retrospective Study. BMC Ophthalmol..

[16] Manolopoulos A., Katsoulas G., Kintos V., Koutsokera M., Lykou C., Lapaki K.M., Acquaviva P.T. (2022). Isolated Abducens Nerve Palsy in a Patient With COVID-19: A Case Report and Literature Review. Neurologist.

[17] Bista B., Yadav R., Gupta S., Das S.S., Rajak A., Acharya R., Neupane R., Bista P.R. (2023). Ocular and Neuro-Ophthalmic Manifestations Post COVID-19 Infection. J. Nepal Health Res. Counc..

[18] Siddiqi A.R., Khan T., Tahir M.J., Asghar M.S., Islam M.S., Yousaf Z., Saranathan M. (2022). Miller Fisher Syndrome after COVID-19 Vaccination: Case Report and Review of Literature. Medicine.

[19] Khor H.D., Lott P.W., Daman Huri S.N.R., Singh S., Iqbal T. (2023). COVID-19 and Crossed Eye: A Case Report and Literature Review. Cureus.

[20] Rethlefsen M.L., Page M.J. (2022). PRISMA 2020 and PRISMA-S: Common Questions on Tracking Records and the Flow Diagram. J. Med. Libr. Assoc..

[21] Su W.-Y., Lu C.-J. (2023). The Clinical Course of New-Onset Ocular Myasthenia Gravis Caused by Pfizer–BioNTech COVID-19 Vaccine. Acta Neurol. Taiwanica.

[22] Hoshina Y., Sowers C., Baker V. (2022). Myasthenia Gravis Presenting after Administration of the MRNA-1273 Vaccine. Eur. J. Case Rep. Intern. Med..

[23] Abicic A., Sitas B., Adamec I., Bilic E., Habek M. (2022). New-Onset Ocular Myasthenia Gravis After Booster Dose of COVID-19 Vaccine. Cureus.

[24] Choi S.Y., Choi J.H., Oh E.H., Choi K.D. (2023). Sequential Orbital Apex Syndrome Following the COVID-19 Vaccination: A Case Report. eNeurologicalSci.

[25] Nanatsue K., Takahashi M., Itaya S., Abe K., Inaba A. (2022). A Case of Miller Fisher Syndrome with Delayed Onset Peripheral Facial Nerve Palsy after COVID-19 Vaccination: A Case Report. BMC Neurol..

[26] Pappaterra M.C., Rivera E.J., Oliver A.L. (2023). Transient Oculomotor Palsy Following the Administration of the Messenger RNA-1273 Vaccine for SARS-CoV-2 Diplopia Following the COVID-19 Vaccine. J. Neuroophthalmol..

[27] Signa S., Brolatti N., Trincianti C., Tortora D., Saffioti C., Di Marco E., Acquila M., Amadori E., Fiorillo C., Ricci E. (2022). Pediatric SARS-CoV-2-Related Diplopia and Mesencephalic Abnormalities. Neurol. Clin. Pract..

[28] Zayet S., Mihoubi A., Chatain M., Sreiri N., Trimech M.B., Gendrin V., Benjelloun F., Klopfenstein T. (2023). Clinical Spectrum of Ocular Manifestations in COVID-19: A Case Series. Infect. Med..

[29] de Medeiros A.L., Martins T., Kattah M., Soares A.K.A., Ventura L.O., Ventura C.V., Barros E. (2022). Isolated Abducens Nerve Palsy Associated with Coronavirus Disease: An 8-Month Follow-Up. Arq. Bras. Oftalmol..

[30] Vasanthapuram V.H., Badakere A. (2021). Internuclear Ophthalmoplegia as a Presenting Feature in a COVID-19-Positive Patient. BMJ Case Rep..

[31] Tremblay C., Brace M. (2023). Treatment of Acquired Partial Oculomotor Nerve Palsy with Dexamethasone—A Case Report. Int. J. Surg. Case Rep..

[32] Hu K., Patel J., Swiston C., Patel B.C. (2025). Ophthalmic Manifestations of Coronavirus (COVID-19).

[33] Dunai C., Collie C., Michael B.D. (2022). Immune-Mediated Mechanisms of COVID-19 Neuropathology. Front. Neurol..

[34] Iwasaki M., Nishizawa T., Iida E., Arioka H. (2023). Third Cranial Nerve Palsy Due to COVID-19 Infection. BMJ Case Rep..

[35] Tan Y.J., Ramesh R., Tan Y.H., Tan S.M.L., Setiawan S. (2023). COVID-19 and Isolated Oculomotor Nerve Palsy: Clinical Features and Outcomes. Clin. Neurol. Neurosurg..

[36] Langford B.J., So M., Raybardhan S., Leung V., Soucy J.P.R., Westwood D., Daneman N., MacFadden D.R. (2021). Antibiotic Prescribing in Patients with COVID-19: Rapid Review and Meta-Analysis. Clin. Microbiol. Infect..

[37] Homik J., Suarez-Almazor M.E., Shea B., Cranney A., Wells G.A., Tugwell P. (1998). Calcium and Vitamin D for Corticosteroid-Induced Osteoporosis. Cochrane Database Syst. Rev..

